# Development of new 9-ball test protocols for assessing expertise in cue sports

**DOI:** 10.1186/s13102-021-00237-9

**Published:** 2021-02-02

**Authors:** Jing Wen Pan, John Komar, Pui Wah Kong

**Affiliations:** 1 Physical Education and Sports Science Academic Group, National Institute of Education, Nanyang Technological University, 1 Nanyang Walk, Singapore, 637616 Singapore; 2 Office of Graduate Studies and Professional Learning, National Institute of Education, Nanyang Technological University, 1 Nanyang Walk, Singapore, 637616 Singapore

**Keywords:** Error, Variability, Kinovea, Playing level, Pool, Billiards

## Abstract

**Background:**

This study aimed to develop new test protocols for evaluating 9-ball expertise levels in cue sports players.

**Methods:**

Thirty-one male 9-ball players at different playing levels were recruited (recreational group, *n* = 8; university team, *n* = 15; national team, *n* = 8). A 15-ball test was administered to indicate overall performance by counting the number of balls potted. Five skill tests (power control, cue alignment, angle, back spin, and top spin) were conducted to evaluate specific techniques by calculating error distances from pre-set targets using 2D video analysis.

**Results:**

Intra-class correlation analyses revealed excellent intra-rater and inter-rater reliability in four out of five skill tests (ICC > 0.95). Significant between-group differences were found in 15-ball test performance (*p* <  0.001) and absolute error distances in the angle (*p* <  0.001), back spin (*p* = 0.006), and top spin tests (*p* = 0.045), with the recreational group performing worst while the national team performing best. Greater inter-trial variability was observed in recreational players than the more skilled players (*p* <  0.005).

**Conclusions:**

In conclusion, the 9-ball test protocols were reliable and could successfully discriminate between different playing levels. Coaches and researchers may employ these protocols to identify errors, monitor training, and rank players.

**Supplementary Information:**

The online version contains supplementary material available at 10.1186/s13102-021-00237-9.

## Background

Nine-ball is one discipline of cue sports playing with a cue stick on a rectangular pool table with six pockets. In the game of 9-ball, there are one cue (white) ball and nine colored balls numbered from 1 to 9. Players should always strike the ball numbered lowest, and the frame winner is the one who legally pots the 9 ball. Internationally, the primary governing body of 9-ball is World Pool-Billiard Association (WPA, https://www.wpa-pool.com/). Professional players with a valid WPA Players license are ranked based on the points they gain in the tournaments such as China Open, All Japan, and 9-Ball World Championships (https://wpapool.com/ranking/). However, recreational and novice players who are not registered with WPA and have not attended the tournaments recognized by WPA are not eligible for the official ranking. Currently, there are no standard tests available to evaluate the expertise levels of cue sports players.

In the literature, studies have described specific cue sports techniques performed by professional players to provide a reference for coaches and players [1–3]. For example, Kornfeind and co-workers [1] conducted a kinematical study of 18 shots including draw shot (back spin), follow shot (top spin), and breaks on professional players who were among the top 80 of the European Pocket Billiard Federation. Similarly, Talts and colleagues [2] reported the muscle activation of 20 elite novus (closely related to pocket billiards) players during the cueing movement. In another study on snooker players [3], participants were divided into groups based on their maximum break (scores accumulated in one consecutive visit). This study used various tests to evaluate essential skill elements of snooker, including power control, angle, top spin, back spin, side spin, and cue alignment. Their video analysis results showed that the tests could effectively differentiate snooker players’ expertise levels. In contrast to recruiting expert players, Haar et al. [4, 5] examined the learning process of a single pool task in 30 beginners who have very little or no experience in playing pool or snooker. To the best of our knowledge, no studies have reported any testing protocol to comprehensively assess expertise in 9-ball which is played under different rules on a smaller table than that of snooker.

Development of standard test protocols is important for expertise evaluation and player selection. In other sports, for instance, a reaction agility test that was designed for elite junior Australian football players could differentiate among players of diverse competition levels [6]. In soccer, Kutlu and colleagues [7] implemented a new test for agility and skill in order to identify players’ talent. For curling players, a novel sport-specific balance test was developed to examine their performance levels [8]. In gymnastics, Khong and co-workers [9] proposed a simple 2D video analysis of the cartwheel movement to effectively discriminate between highly trained gymnasts and novices. These studies on various sports suggest that using basic skill tests incorporated with 2D video analysis are plausible means to develop specific 9-ball test protocols.

The primary purpose of this study was to develop new test protocols with 2D video analysis for evaluating expertise in 9-ball. A secondary purpose was to establish the intra-rater and inter-rater reliability of the new tests developed. It was hypothesized that the new test protocols would effectively distinguish cue sports players of various playing levels and demonstrate high reliability in the video analysis.

## Methods

This study adopted a cross-sectional comparative design. Participants of various playing levels were required to complete a 15-ball performance test as well as five skill tests specific to 9-ball which were developed based on a previous study on snooker [3]. Intra-rater and inter-rater reliability were tested for the data extracted from the videotapes. The performance of each test was compared among participants of different playing levels.

### Participants

The present study was approved by the Nanyang Technological University Institutional Review Board (Protocol Number: IRB-2019-05-013). All methods of this study were performed in accordance with the Declaration of Helsinki. Thirty-one male participants [mean (standard deviation); 26.0 (6.5) years old; height 172.5 (5.7) cm; body mass 69.6 (12.2) kg; 3 left-handed and 28 right-handed] provided written informed consent to participate (Table 1). Parental consent was also obtained for minor participants who were under 21 years old. The participants were 9-ball players recruited from the national cue sports team of Singapore, university team of Nanyang Technological University and Singapore Management University, and recreational population. Participants were surveyed about their playing experience which was defined as the number of years they had been regularly playing 9-ball or 8-ball. The inclusion criteria were that participants 1) had at least 1 year of playing experience, and 2) were currently active 9-ball players. Participants would be excluded if they 1) had any upper body injuries for 3 months prior to the study, 2) had any history of shoulder, elbow, wrist, or hand surgery, or 3) were experiencing pain or discomfort in any part of the body when playing 9-ball at the time of this study.

**Table 1:** Demographic and anthropometric characteristics of participants

	Recreational (R) (*n* = 8)	University (U) (*n* = 15)	National (N) (*n* = 8)	*P*	*Post-hoc*	
Age [years]	26.4 (1.1)	24.1 (2.5)	29.1 (12.2)	0.028*	U < R	
Experience [years]	2.0 (1.9)	5.1 (3.5)	12.1 (9.1)	0.007*	R < U	R < N
Height [cm]	171.6 (5.2)	173.0 (5.5)	172.4 (7.3)	0.867		
Body mass [kg]	70.9 (8.9)	66.9 (13.9)	73.4 (11.5)	0.464		

Significant difference (*p* <  0.05) is shown in bold text and indicated by an asterisk

### Design and procedures

The recreational group and university team completed the experiment session on one pool table at Nanyang Technological University; the national team was tested on another table in their usual training venue. Both tables were 9-ft 9-ball tables with the same dimension (height 80 cm; playing surface 254 cm × 127 cm). Thirteen out of 31 participants did not bring their own cue stick and used a standard one (full length 147 cm; mass 591 g) provided by the research team. The length and mass of the cue sticks for participants who brought their own sticks were 147.2 [147.0, 147.5] cm and 566.9 [558.0, 575.8] g, respectively. Individual difference of the cue sticks was considered negligible as the length and mass were largely similar. A set of pool balls (diameter 5.72 cm, Cyclop ZEUS Tournament TV set, Xinzhan Co., LTD, Shanghai, China) were used for both warm-up and experiment sessions. A digital camera (30 Hz, model EX-100, Casio Computer CO., LTD, Tokyo, Japan) was used to videotape the ball movements on the pool tables from a top view.

**Fig. 1:**
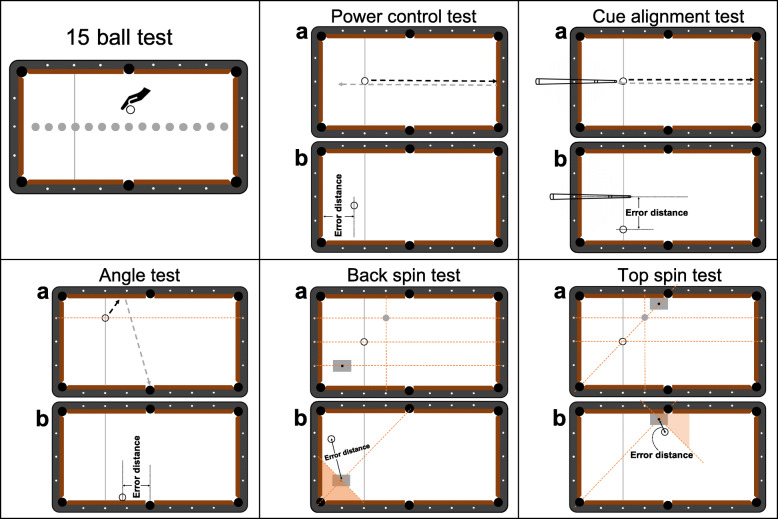
Schematic representation of the 15-ball test and five skills tests

Prior to data collection, participants were required to warm up (e.g. potting balls freely, potting balls with back or top spin applied) on the experiment pool tables for approximately 10 min. They were then instructed to conduct a 15-ball test which is widely employed by coaches and athletes during regular 9-ball training (Fig. 1 ‘15-ball test’). This 15-ball test was set to evaluate players’ overall performance as it could reflect players’ capability of potting balls accurately and positioning cue ball for subsequent shots. In this performance test, 15 object balls were lined up in the center of the pool table and the cue ball (ball in hand) was placed by participants anywhere on the table in the beginning of the test. Participants were required to pot as many balls as possible in one consecutive visit in no particular order. The number of balls successfully potted in one visit was counted (maximum 15 balls) to indicate performance. A total of three trials were administrated for each participant, and the sum of the best two trials (with more balls potted, and hence maximum 30 balls) were used for analysis.

To test participants’ specific skills, five tests were developed to cover the key skill components of the 9-ball game. These tests were modified from previous study on snooker [3], including: 1) power control, 2) cue alignment, 3) angle, 4) back spin, and 5) top spin tests. Participants performed 10 valid trials for each test [3].

#### Power control test

This test was developed to simulate the lagging shot in 9-ball. In many 9-ball tournaments, a lag is officially applied to determine the order of play, and the winner of the lagging shot is the one who positions the ball closer to the top cushion (smaller error distance) after bouncing off the bottom cushion. The winner of a lagging shot can choose to take the first play and take advantage of it. Power control is also a basic aspect in every shot in cue sports. Potting a ball with specific amount of power allows the player to position the cue ball after shots, and/or to escape a snooker whereby the object ball is blocked by other balls. In the power control test, participants stroke a cue ball, which was placed at the center of the head string, down the table (Fig. 1 ‘Power control test’ a). The cue ball was expected to stay at the top cushion after bouncing off the bottom cushion, and the error distance was considered 0; otherwise, an error distance between the top cushion and the center of the cue ball was measured (Fig. 1 ‘Power control test’ b). While we did not directly measure the power in this ‘power control test’, as previously done by Chung and co-workers [3], we associated the power with the end distance of the ball from the target. If ‘too much’ power is used, the ball would have touched the top cushion and then bounced back, resulting in a positive error distance. Likewise, a negative error distance would mean ‘not enough’ power as the cue ball does not travel far enough to touch the top cushion. It was counted as a valid trial no matter the cue ball touched the top cushion or not.

#### Cue alignment test

This test was designed to evaluate the ability of striking straight, which is also an essential skill in 9-ball. If one strikes the cue ball perpendicular to the bottom cushion, the ball will return in the same straight line, and ideally, the cue ball will touch the cue tip. This touch-tip style is widely implemented in training which gives players direct feedback on whether the ball is stroke in a straight line. In the current study, a cue ball was placed at the center of the head string (Fig. 1 ‘Cue alignment test’ a). Participants drove the cue ball towards the bottom cushion and expected it to return and touch the cue tip. The error distance was measured if the cue ball did not touch the cue tip (Fig. 1 ‘Cue alignment test’ b). It was considered negative when the cue ball was at the right side on the pool table and positive when cue ball was at the left side.

#### Angle test

Understanding the path of the ball is necessary, especially for escaping a snooker when the object ball is blocked by other balls. When striking the cue ball to the cushion, the bounce off angle (angle of reflect) is equal to the angle of incidence if no spin is applied. To test this ability, the current study asked participants to drive a cue ball to the middle pocket after bouncing off the opposite cushion. This simple task clearly informed the participants if the cue ball was driven in the correct path. In this angle test, a cue ball was set in the head string aligning the second diamond of the top cushion (Fig. 1 ‘Angle test’ a). The player should aim to strike the cue ball to the side cushion and expected it to fall into the opposite middle pocket. An error distance was measured between the center of the cue ball and the center of the pocket if the cue ball did not fall into the middle pocket (Fig. 1 ‘Angle test’ b). The error distance was considered negative if the cue ball ended close to the head string and positive when the end position of the cue ball was near the bottom cushion.

#### Back spin test

Back spin is employed very often in order to position the cue ball after hitting an object ball. It calls for precision to make the cue ball finishing at a ‘good’ position, preparing for the next shot. Hence, this test instructed participants to draw the cue ball back to a specific target with appropriate back spin applied. A cue ball was placed at the center of the head string and an object ball was set in alignment with the second diamond of the top cushion and the fourth diamond of the side cushion (Fig. 1 ‘Back spin test’ a). Participants were required to pot the object ball and draw the cue ball back. The cue ball was expected to return to a target area which was represented by a quarter of a piece of a A4 sized paper (7.5 cm × 5.3 cm), and the center of the target was in line with the fourth and second diamond of the top and side cushion, respectively. Only shots with the object ball potted were counted as valid trials; otherwise, the participants were asked to pot again. The error distance was measured between the end position of the cue ball center and the target center (the black dot, Fig. 1 ‘Back spin test’ b). When the back spin was ‘not enough’ and the cue ball ended in front of the target center, the error distance was considered negative. The error distance would be positive if the back spin was ‘too much’ such that the cue ball passed target center and stayed in the shaded area.

#### Top spin test

Similar to back spin, top spin is also used frequently to position the cue ball for the subsequent shot. To test one’s ability to generate top spin, the test requires the cue ball to continue traveling after potting an object ball. The cue ball and object ball were set in the same way as the back spin test and the same target (7.5 cm × 5.3 cm) was placed near the middle pocket (Fig. 1 ‘Top spin test’ a). Participants were asked to pot the object ball with top spin and drive the cue ball to the center of the target. A negative error distance was measured when top spin was ‘not enough’ and the cue ball could not reach the center of the target (Fig. 1 ‘Top spin test’ b). A positive error distance was measured when ‘too much’ top spin was applied such that the cue ball passed the target center and stayed in the shaded area.

### Video data processing

Videotapes for the five 9-ball skill tests were analyzed using a free software Kinovea (version 0.8.27, available for download at: http://www.kinovea.org). In the software, the tool ‘perspective grid’ was used to calibrate the pool table, wherein only the half table was used as the end position of the cue ball as well as the target were on this side of the pool table. The perspective grid was then set at the dimension of 127 cm × 127 cm which was same as the actual playing surface. The error distance was measured accordingly between the target and the center of the cue ball for each test.

The error distances of all trials were firstly processed by one assessor (JWP). To test the intra-rater reliability, a sub-sample of the data which were the first valid trial of each test per participant (5 tests × 31 participants = 155 trials) was measured again by the same assessor (JWP) 1 month apart. To assess inter-rater reliability, the sub-sample of data were also independently measured by a second assessor (MJC).

Within each participant, it is possible that both positive and negative error distances co-exist among the 10 trials. The positive and negative values can potentially cancel out each other if we simply take an average value of all 10 trials. To resolve this issue, we proposed two types of variables in our analysis. First, to assess the overall performance for a test, an absolute mean error distance was calculated as the mean value of the absolute error distances from 10 trials due to the possibility that both positive and negative error distances co-exist among the 10 trials. This absolute mean error distance indicates the magnitude of the error across the 10 trials regardless of the error direction. Second, to provide more specific feedback, it is also important to distinguish whether a shot is ‘too long’ or ‘too short’, and whether the spin is ‘too much’ or ‘not enough’. Thus, a positive mean error distance was calculated from all positive values within the 10 trials. Likewise, a negative mean error distance was calculated from all negative values within the 10 trials. If a participant did not have any positive or negative error distances in the 10 trials, the corresponding mean error distance was left blank. For each participant, the standard deviation (SD) of the 10 trials was also obtained to reflect the inter-trial variability of the error distances within an individual [10, 11].

### Statistical analysis

Statistical analysis was performed using SPSS (version 26.0, IBM Corp, Armonk, USA). For the 15-ball performance test, the sum of number of balls potted in the best two trials was used for each participant. For the 2D video analysis, intra-rater reliability for the first assessor (two sets of measurements) and inter-rater reliability (first set of measurement of each assessor) were assessed using intraclass correlation coefficients (ICC_2,1_ and ICC_2,2_, respectively). Interpretation of reliability results were interpreted as poor (ICC <  0.50), moderate (0.5 ≤ ICC <  0.75), good (0.75 ≤ ICC <  0.9), or excellent (0.9 ≤ ICC) [12].

A one-way analysis of variance (ANOVA) was conducted to evaluate the group differences among the recreational group, university team, and national team in the 15-ball performance test and the five 9-ball skill tests (error distances, inter-trial variabilities). The assumption of homogeneity of variances was tested using Levene’s test; Welch test was applied when the assumption of homogeneity of variances was violated. A Tukey HSD *post-hoc* test was performed accordingly; a Games-Howell test was used instead when the assumption of homogeneity of variances was violated. ANOVA effect size (partial eta-squared, η_p_^2^) was interpreted as small (η_p_^2^ = 0.01), medium (η_p_^2^ = 0.06), or large (η_p_^2^ = 0.14) [13]. The associations between the results of the 15-ball test and the five skill tests were assessed using Spearman’s rho (r_s_). Spearman correlation coefficient (r_s_) was interpreted as negligible (0 ≤ |r_s_| < .30), low (.30 ≤ |r_s_| < .50), moderate (.50 ≤ |r_s_| < .70), high (.70 ≤ |r_s_| < .90), or very high (.90 ≤ |r_s_| ≤1.00) [14]. All statistical significance was set at the 0.05 level.

## Results

Both ICC_2,1_ and ICC_2,2_ analyses for the four skill tests (power control, cue alignment, angle, and back spin) were greater than 0.95, indicating excellent intra-rater and inter-rater reliability. The top spin test showed moderate reliability for intra-rater test (ICC = 0.68) and good reliability for inter-rater assessment (ICC = 0.75).

The 15-ball test performance significantly differed among the three groups (*p* <  0.001, Table 2), with the recreational group potted the fewest balls and national team potted the most. For the 9-ball skill tests, the absolute error distances were greater in the recreational group compared with the other two groups in the angle (*p* < 0.001), back spin (*p* = 0.006), and top spin tests (*p* = 0.045). Positive error distance of the angle test was found to be greater (*p* < 0.001) in the recreational group than the other two groups; similar finding was revealed in the negative error distance of the back spin test (*p* = 0.010). Difference in between the national and university teams was only found in the absolute error distance of the angle test.

**Table 2:** Comparison of the 15-ball test performance and error distances of the five 9-ball skill tests among players of different playing levels

	Recreational (R) (*n* = 8)	University (U) (*n* = 15)	National (N) (*n* = 8)	*P*	η_p_^2^	*Post-hoc*		
Balls potted	4.8 (2.2)	11.3 (4.7)	20.6 (6.4)	< 0.001*	0.218	R < U	R < N	U < N
Power control [cm]								
Absolute error	44.7 (16.7)	32.9 (9.0)	28.7 (6.0)	0.060	0.257			
Positive error	42.0 (18.8)	27.4 (10.2)	25.0 (8.0)	0.106	0.247			
Negative error	−48.2 (23.0)	− 38.7 (16.6)	−31.5 (11.4)	0.176	0.121			
Cue alignment [cm]								
Absolute error	6.3 (4.8)	4.5 (2.6)	2.3 (1.7)	0.053	0.189			
Positive error	2.8 (1.9)	2.9 (1.7)	2.5 (0.9)	0.958	0.006			
Negative error	−6.0 (6.1)	−4.4 (2.7)	−2.3 (1.7)	0.233	0.119			
Angle [cm]								
Absolute error	8.0 (1.5)	4.8 (2.7)	2.3 (1.1)	< 0.001*	0.517	R > U	R > N	U > N
Positive error	7.0 (1.9)	4.0 (2.2)	2.0 (1.4)	< 0.001*	0.504	R > U	R > N	
Negative error	−5.0 (3.1)	−3.5 (2.4)	−1.4 (0.5)	0.080	0.205			
Back spin [cm]								
Absolute error	64.9 (24.2)	26.5 (5.2)	26.4 (5.0)	0.006*	0.662	R > U	R > N	
Positive error	28.4 (3.1)	20.2 (5.3)	18.0 (6.1)	0.079	0.214			
Negative error	− 65.1 (24.0)	− 29.6 (7.0)	−30.0 (6.1)	0.010*	0.605	R > U	R > N	
Top spin [cm]								
Absolute error	15.5 (5.7)	10.3 (3.8)	10.7 (3.3)	0.045*	0.219	R > U		
Positive error	9.7 (7.6)	8.0 (2.3)	6.0 (2.8)	0.297	0.110			
Negative error	−15.3 (7.4)	−11.5 (5.0)	−11.6 (4.3)	0.342	0.082			

Balls potted refers to the number of balls potted in the 15-ball test (sum of best 2 out of 3 trials). Significant difference (*p* < 0.05) is shown in bold text and indicated by an asterisk. An absolute error was calculated as the mean value of the absolute error distances from 10 trials; a negative mean error distance was calculated from all negative values within the 10 trials; a positive mean error distance was calculated from all positive values within the 10 trials

**Fig. 2:**
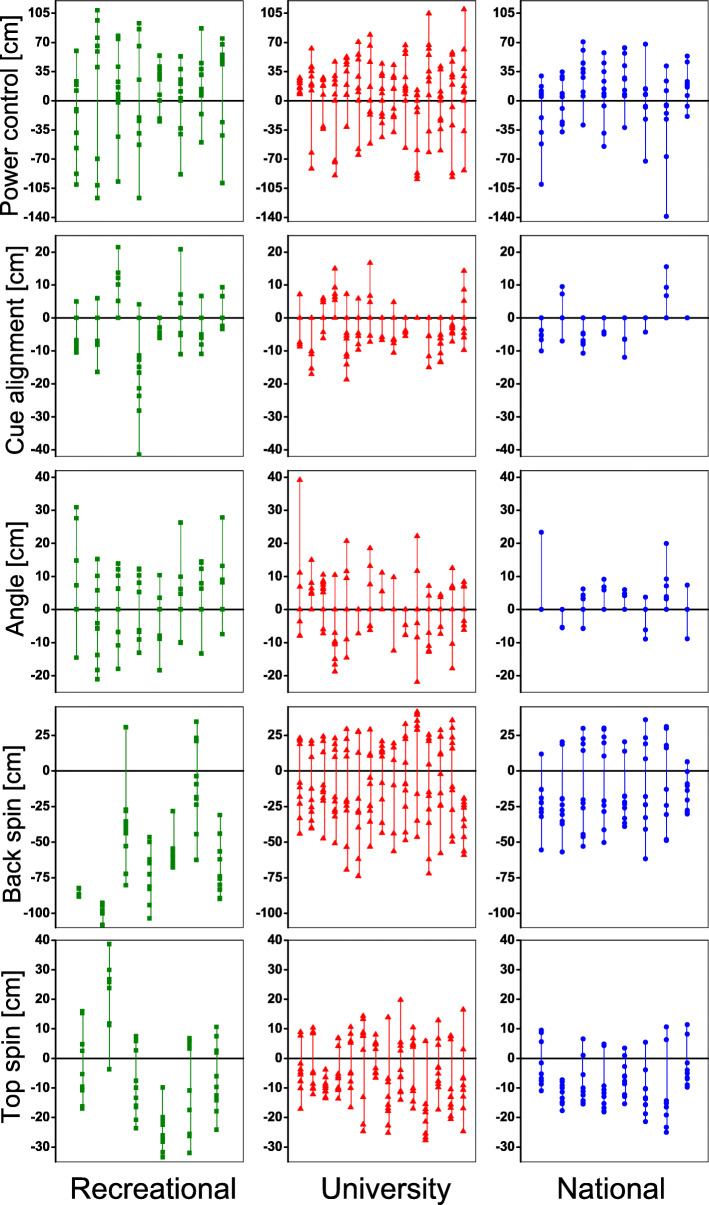
Error distances of 10 individual trials for each participant. The recreational group is shown in green squares; the university team is shown in red triangle; the national team is shown in blue dot

There was considerable intra-individual variability of the error distances among the 10 trials within players (Fig. 2). In all except the back spin test, greater variability of the error distances was observed in the recreational group compared with either the national or the university team (Table 3). For the back spin test, the opposite pattern was found whereby the recreational group displayed the highest inter-trial variability. Similar to the error distance results, only the angle test was able to show lower variability in the national team than the university team.

**Table 3:** Inter-trial variability of the error distances of the five 9-ball tests among players of different playing levels

	Recreational (R) (*n* = 8)	University (U) (*n* = 15)	National (N) (*n* = 8)	*P*	η_p_^2^	*Post-hoc*		
Power control [cm]	52.0 (17.8)	37.6 (13.3)	33.5 (9.2)	0.025*	0.231	R > N		
Cue alignment [cm]	6.6 (3.3)	4.7 (2.1)	3.1 (1.9)	0.024*	0.234	R > N		
Angle [cm]	10.5 (1.8)	6.9 (3.3)	4.2 (1.8)	< 0.001*	0.449	R > U	R > N	U > N
Back spin [cm]	16.9 (11.0)	27.9 (5.9)	25.3 (7.5)	0.016*	0.265	R < U		
Top spin [cm]	11.7 (2.8)	9.3 (2.8)	7.7 (2.7)	0.046*	0.218	R > N		

Inter-trial variability was represented by standard deviation of the 10 trials within an individual and mean values (standard deviations) were calculated for each group. Significant difference (*p* < 0.05) is shown in bold text and indicated by an asterisk

**Fig. 3:**
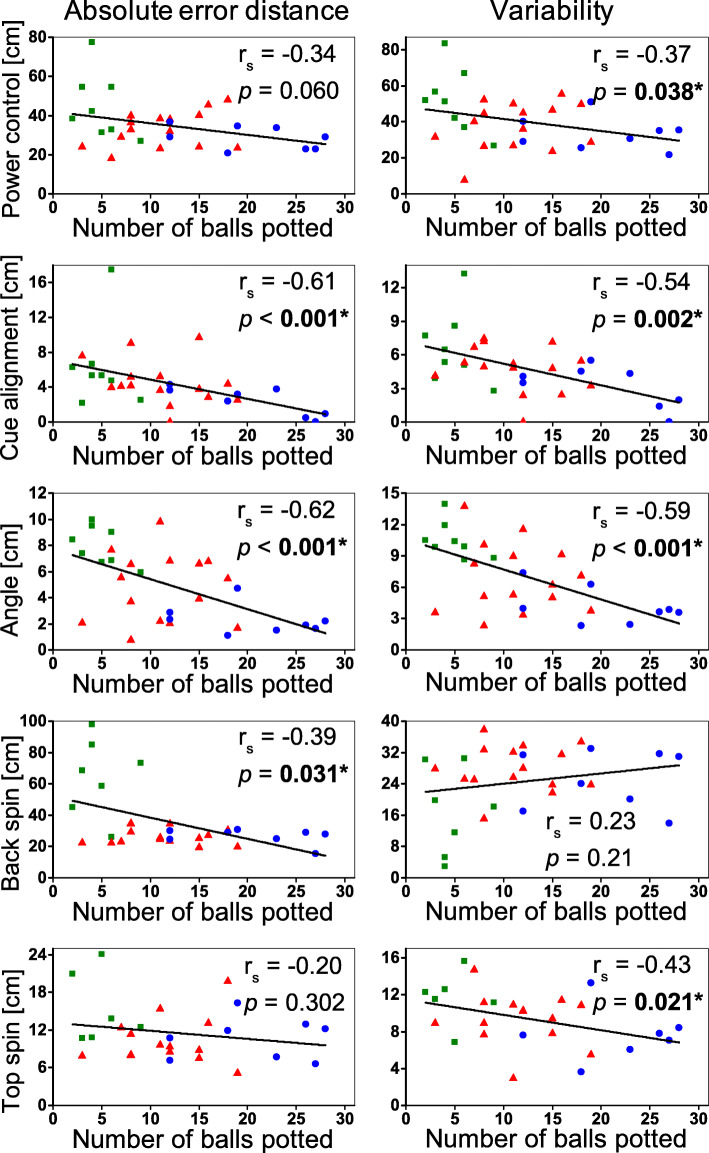
Correlations between 9-ball skill tests (absolute error distance and variability) and the 15-ball performance test (number of balls potted in best 2 out of 3 trials). The recreational group is shown in green squares; the university team is shown in red triangle; the national team is shown in blue dot

There were significant negative correlations between the number of balls potted in the 15-ball test and the absolute error distances of the cue alignment, angle, and back spin tasks (Fig. 3). These results reflected that higher performance level is associated with smaller magnitude of error. In addition, the inter-trial variability in four out of five tests showed significant negative correlations with the 15-ball test performance. These findings indicated that as expertise level increased, cue sports players were more consistent when executing repeated skill tests.

## Discussion

This study presented novel protocols to test the expertise levels of cue sports players in the game of 9-ball. The findings showed that the 15-ball test and the error distances in three out of five specific skill tests could effectively discriminate among players at different playing levels. The inter-trial variability was significantly different among three groups of participants in all five skill tests. The intra-rater and inter-rater reliability of the video analysis were excellent in four out of five skill tests. Additionally, the 15-ball test performance had significant correlations with the error distances and variability of most 9-ball skill tests. Overall, the hypothesis of the present study is partially supported as most tests developed could effectively distinguish players of different playing levels with excellent reliability.

### Error distances of the five 9-ball skill tests

In order to evaluate participants’ specific skill in 9-ball, test protocols were developed that required players to demonstrate their control of power and spin and understanding of the ball path. Similar to previous studies on gymnastics which applied video analyses to evaluate gymnasts’ ability [9, 15], the current study also found excellent intra-rater and inter-rater reliability for most 9-ball skill tests analyzed using simple 2D video analysis. By measuring the error distances from pre-set targets, three out of the five skill tests were effective in discriminating among participants at varied expertise levels in 9-ball. This finding is in line with a previous study on snooker which used skill tests to differentiate players of varied abilities [3]. Since the five skill tests developed are simple to administer and only require one video camera, it is plausible for cue sports coaches and researchers to adopt our protocols. For practical application, one can easily conduct the tests in pool clubs and training venues to evaluate players’ performance on specific skills. The present study demonstrated that specific cue sports performance can be objectively quantified by error distances from pre-set targets using 2D video analysis. This approach can be useful in screening and talent identification, monitoring players’ progression, and implementing appropriate training regimes.

Generally, the recreational group had the largest error distances from the pre-set target while the national team had the smallest errors (Table 2). This finding is not surprising as ‘skilled performance’ can be defined as the ability to complete precise movements or achieve reset goal [16]. The five tests developed in this present study were built upon a previous study on snooker [3] to fit the equipment and rules of 9-ball. For instance, the power control test was to resemble the lagging shot in actual 9-ball games. In the current study, the error distances of the power control test did not differ among the three groups, although the results of approaching statistical significance (*p* = .060). For the cue alignment test which was designed to evaluate whether a player could strike in a straight direction, this study observed a borderline (*p* = .053) significance among groups. Hence, these two skill tests are considered not sensitive enough for distinguishing between expertise levels.

The other three skill tests (angle, back spin, and top spin tests) were shown to effectively classify participants as the means of error distances were greater in the recreational players than the more proficient players. In the angle test, both the national team and university team drove more balls into the pocket (i.e. zero error distance) than the recreational group. Most importantly, only the angle test statistically showed that the national team performed better with smaller error distances than the university team according to the post hoc analysis (Table 2). This indicated that the angle test was the most sensitive test among all five specific skill tests to identify the ‘best’ from the ‘good’ players.

The back spin test may be the most challenging among these five skill tests [3]. When striking balls with back spin, appropriate amount of back spin was required to place the cue ball at a specific target. In the present study, the recreational players struggled to apply sufficient back spin and hence, the cue ball tended to be ‘too far’ (large negative error distance) from the target (Table 2, Fig. 2). Their error distances were much greater than those of the university team and national team. These results reflected that the back spin test was effective in differentiating between average and proficient players but unable to further distinguish the subtle differences within expert players. For the top spin test, the target was set near the middle pocket, posing challenges for players because they were not allowed to pocket the cue ball. While this test also successfully discriminated participants’ expertise levels between the recreational and university teams, it cannot identify the ‘best’ from the ‘good’ players. There are a few possible reasons for why the back spin and top spin tests cannot discriminate between the university team and national team. First, successful 9-ball performance could be determined by multiple factors such as good skills, smart strategy, and effective stress management under pressure. The tests developed in the present study primarily targeted the technical skill proficiency in cue sports; other tactical and psychological factors were not considered. Second, it is possible that the back spin and top spin tests are considered rather ‘easy’ for both university and national players who were very experienced and have mastered the skills.

### Variability in the five 9-ball skill tests

The inter-trial variability was significantly different among three groups of participants in all five skill tests developed in the present study (Table 3). This finding indicated that skilled participants were capable of conducting shots more consistently compared with less skilled players, which is in good agreement with a previous study on snooker [3]. In other studies on human movement, greater variability of the error is considered to represent less desirable performance [17, 18]. For example, higher gait asymmetry between the left and right legs is associated with reduced gait stability [17]. Skilled golfers were found to exhibit higher stability (lower variability) in performance than non-skilled players [18]. In general, small inter-trial variability is considered a sign of good performance as the players are able to strike balls consistently. This pattern was observed in four out of five specific 9-ball skill tests in the present study whereby players at higher playing levels exhibited low inter-trial variability and hence more stable performance. However, in the back spin test, lower variability was found in the recreational group compared with the university team. This result did not imply that the recreational players performed well in their back spin control. On the contrary, recreational players were unable to draw the cue ball back by applying sufficient back spin due to their low expertise level. They could merely stop the cue ball upon striking the object ball, as reflected by their consistently large and primarily negative error distances (Fig. 2). Therefore, the back spin test may not be suitable for novice or recreational players who cannot conduct shots with sufficient back spin applied. Compared with mean error distances, results from the present study suggested that inter-trial variability may be even more effective to discriminate between different expertise levels in cue sports.

### Fifteen-ball performance test

The 15-ball test is a practice drill that is widely utilized in training to develop players’ skill in potting the object balls and positioning the cue ball for subsequent shot. In this test, the cue ball should be continuously set at a ‘good’ position in preparation for the next shot. Different from the five skill tests developed in the present study, the 15-ball test represents players’ overall performance level without pinpointing to any specific skill. In the game of 9-ball, players are required to pot balls and eventually make the 9 ball pocketed legally. The 15-ball test performance was shown to well reflect cue sports players’ general performance as the number of balls potted significantly differed among all three groups of players (Table 2). This finding is promising because the 15-ball test is easy to implement by simply counting the number of balls potted in one visit with no additional equipment or video analysis required. For the purpose of screening a cue sports player’s overall performance, the simple 15-ball test is highly recommended. When feedbacks on specific aspects of 9-ball skills (e.g., power and spin control) are required, coaches can supplement the 15-ball test with specific skill tests such as those developed in the present study.

To perform well in the 15-ball test, cue sports players are likely proficient in many skills such as the control of power, ball path, angle, and spins. Thus, correlation analyses were performed to examine the relationships between 15-ball test performance and the five specific skill tests. The results showed significant negative correlations between the number of balls potted in the 15-ball test and the absolute error distances of three skill tests (cue alignment, angle, and back spin tests, Fig. 3). Similarly, the performance of the 15-ball test was negatively correlated to the inter-trial variability in four out of five tests (power control, cue alignment, angle, and top spin tests, Fig. 3). The angle test represents the understanding and control of complex ball trajectory, and the ability to exhibit consistency in performance. As both skill tests  showed very strong correlation with the performance in the 15-ball test, those specific skills appeared as good indicators of high skill level alongside the performance in the 15-ball test. The lack of significant correlation in variability of the back spin test was likely because that the recreational players were unable to execute the back spin shots. These correlation results suggested that while 15-ball test evaluated a player’s overall ability, this test can reflect specific skills reasonably well especially for cue alignment and angle control. Practically, the simple 15-ball test can be conducted in a very short period of time to evaluate an individual’s performance level with no additional equipment, coach, judge, or video analysis. By counting the number of balls potted in the best two out of three trials, one can rank a group of players of varied ability. Coaches and administrators can consider this test to facilitate the team selection and talent identification process. Players can self-administer the test to get easy and instant feedback on their overall performance levels. Researchers can apply this test to divide participants into groups and/or determine eligibility for participation. Collectively, the 15-ball test is an effective and simple for evaluating 9-ball players’ overall performance levels. While the simple 15-ball test may partially reflect players’ expertise level in various 9-ball skills, coachers are recommended to obtain more feedback using specific skill tests such as those on power and spin control.

### Limitations

There are several limitations to the current study. Firstly, this study was conducted in two venues with different pool tables for convenience. Although the sizes of the two tables were same, some subtle differences (such as different tablecloths) may have influenced the results. Secondly, this study applied a piece of paper to work as a target for the back spin and top spin tests. The physical presence of the paper on the pool table may have affected the final trajectory of the cue ball. If possible, future studies are advised to use other methods such as laser projection or drawing to set targets on the pool table. It should be noted that this study solely recruited male 9-ball players due to the limited number of eligible female players available in Singapore. Hence, the test results may not be directly applicable to female players. In the future, a bigger sample size with both male and  female players should be included to confirm the present findings. Lastly, most skill tests, except the angle test, failed to identify the ‘best’ from the ‘good’ players. Thus, these tests are only appropriate for discriminating between less experienced and experienced players, while unable to detect the subtle differences among experienced players.

## Conclusions

This study presented new test protocols consisted of a 15-ball test for overall performance and five specific 9-ball skill tests to evaluate the expertise levels of cue sports players. Using one camera and simple video analysis, error distances measured in three (angle, back spin, and top spin tests) out of the five specific skill tests were found to successfully discriminate between proficient and less skilled players. Only the angle test can successfully identify the ‘best’ from the ‘good’ players. Inter-trial variability which represents the shot consistency was effective in differentiating between expertise levels, in particular for the angle test. While the present study described test protocols specific for 9-ball skills, coaches and researchers may modify the protocols for other games such as 8-ball since the key techniques and elements of cue sports are similar across many sub-disciplines. With no additional equipment or analysis needed, the simple 15-ball test is an excellent tool to evaluate player’s overall performance as it was effective in discriminating among different performance levels and the performance was well correlated with specific 9-ball skill tests. Coaches, administrators, and researchers may employ this 15-ball test to rank a group of participants by simply counting the number of balls potted. When more detailed feedbacks on key 9-ball skills are required, specific skill tests should be employed to pinpoint the areas for improvement.

## Supplementary Information

**Additional file 1.**

## Data Availability

The datasets used and/or analyzed during the current study are available as supplementary material.
